# Production of Erythropoietin-Specific Polyclonal Antibodies

**DOI:** 10.15171/ijb.1413

**Published:** 2017-03

**Authors:** Kourosh Maboudi, Seyedeh Marzieh Hosseini, Mina Sepahi, Hashem Yaghoubi, Shahin Hadadian

**Affiliations:** ^1^ Department of Biochemistry, Ardabil Branch, Islamic Azad University, Ardabil, 3159915111, Iran; ^2^ Quality Control Department, Pasteur Institute of Iran, Karaj, 31635/157, Iran; ^3^ Recombinant Biopharmaceutical Production Department, Pasteur Institute of Iran, Karaj, 31635/157, Iran; ^4^ Nano-Biotechnology Department, Pasteur Institute of Iran, Tehran,1316943551, Iran

**Keywords:** Polyclonal antibodies, erythropoietin, ELISA, Sensitivity, Specifi city, Protein Precipitation

## Abstract

**Background:**

Erythropoietin, as a principal hormone promotes red blood cell production in bone marrow. Varieties of erythropoietin biosimilar are being produced by recombinant DNA technology in cell cultures. The detection or quantifi cation of these molecules are being performed by diff erent methods which some of theme such as Western blot and enzymelinked immunosorbent assay (ELISA) require specifi c antibodies. High cost, inappropriate shipping (cold chain failures), reduced sensitivity and thus poor detection performance are common pitfalls of using commercial kits for performing immunological tests.

**Objectives:**

To produce in-house polyclonal antibody against active pharmaceutical ingredient (API) of recombinant human erythropoietin (rh-EPO) was the aim of this study.

**Materials and Methods:**

Two healthy female albino rabbits were injected four times in 14 days interval using rh-EPO API as antigen. The produced antibody was separated from plasma via either caprylic acid or saturated ammonium sulfate precipitation and the results were compared from each purification methodologies. The antibody was further purified by ion exchange chromatography. Acceptable purity and good immunogenicity were detected respectively by SDS-PAGE and western blot analysis. The purified antibody was compared with a commercial kit to determine rh-EPO concentration in diff erent steps of production batches via ELISA.

**Results:**

The purity of antibodies after ion exchange chromatography, obtained from caprylic acid and ammonium sulfate precipitation were 97 and 80%, respectively.

**Conclusions:**

As producing in house kits is one of the important challenges of bio- pharmaceutical manufacturers, a simple, cost- and time-effective, and easy to scale up strategy for making in-house polyclonal antibody was set up. Caprylic acid precipitation resulted higher purity than ammonium sulfate and fi nally purified antibody (97% purity) used as a capture antibody in sandwich ELISA test was able to detect erythropoietin antigen as sensitive (100%) and specifi c (100%) as commercial kits.

## 1. Background

Regarding the achievements of pharmaceutical biotechnology during the last two decades, new classes of pharmaceutical compounds have entered the market. Recombinant human Erythropoietin is one of these products (1-3). Erythropoietin is one of the highly used recombinant drugs in the world (4). Glycoprotein molecule weighing 30 kDa and 165 amino acid that helps the growth and differentiation of immature blood cells (5). The hormone is naturally produced by kidneys in response to hypoxia and it is necessary for ensuring red blood cells production in bone marrow. Recombinant human erythropoietin is used for anemia, especially the treatment of anemia related to chronic kidney disease, and HIV (4). The DNA recombinant technology has made the advantages of producing different biosimilars of erythropoietin in different mammalian cell lines (6). Chinese hamster ovary (CHO) cell lines have been used widely in industrial scale production of erythropoietin (7). As like as other biosimilar products, the reproducibility of product quality, safety and efficacy profile with original products, are the matter of concerns (8). To carry out the quality control and sometimes quantity check for recombinant erythropoietin, immunoassay is the method of choice. Specific antibodies can be used in Western or dot blot analysis (qualitative) and enzyme-linked immunoassay (ELISA).

## 2. Objectives

High cost, inappropriate shipping (cold chain failures), reduced sensitivity and thus poor detection performance are common pitfalls of using commercial kits for performing immunological tests. Usually recombinant biopharmaceutical manufactures improve the sensitivity and selectivity by making in house kits for their own and sometimes they do attempt to substitute some parts of commercial kits with in- house counterparts. For example most commercial Sandwich ELISA kits are designed for quantifications of different antigens in picogram concentrations for sera. In the case of using such kits with high concentrations, sample dilution is necessary that causes the risk of manipulation errors.

In this research, active pharmaceutical ingredient (API) of recombinant human erythropoietin produced in Pasteur institute of Iran was used to produce in-house polyclonal antibody.

## 3. Materials and Methods

### 3.1. Antibody Production

Active pharmaceutical ingredient of recombinant erythropoietin (Pasteur Institute, Iran) was selected as the antigen for injection. Freund’s adjuvant (Sigma Aldrich-Germany) was combined with antigen in equal quantities. Two healthy female albino rabbits (Pasteur Institute, Iran) were prepared (chest hair shaved and disinfected with alcohol) and were subcutaneous injected in 10 points (200 µL of 0.35 µg.µL^-1^ rh-EPO antigen in phosphate buffer pH 7.0 and adjuvant), which means 70 µg antigen totally (9) four times with a 14 days intervals using complete Freund’s adjuvant at first injection and incomplete adjuvant as the booster at second to fourth injections (10-13). Working with laboratory animals were performed according to national guidelines issued by The Ministry of Health based on national ethical codes. The rabbits’ bloods were centrifuged (10000 ×g, 10 min). Separated blood sera were pooled and stored at -20°C (13).

### 3.2. Antibody- Antigen Interaction Assays

#### 3.2.1. Agglutination

To assay for the presence of EPO antibody, the centrifuged serum droplets (2, 5, 10, 20, 40 and 80 µL) were put on a glass slide. Antigen (30 µL EPO) was added to each slide and the positive or negative agglutinations were checked by naked eyes (14).

#### 3.2.2. Ouchterlony

Ouchterlony’s gel diffusion method in plates was used to check specific antibody- antigen reactions (15). Agarose solution (1% w/v) containing 0.15 M NaCl (Merck Millipore,Germany) was melted and poured on a slide to make a 3 mm thick layer. Several separated holes were punched on gel surface and different antigens including API of interferon, hepatitis B surface antigen, streptokinase and erythropoietin (all from Pasteur institute, Iran) were added to the holes and the slide was put in a Petri dish plate at 22°C. Symmetry of precipitate, archline formation, indicating the antibody- antigen interaction was checked by naked eyes.

### 3.3. Separation of Immunoglobulin

Caprylic acid and ammonium sulfate were separately used for purification of the antibody and the outcomes were compared. In caprylic acid method, 30 mL of 60 mM sodium acetate solution (Merck Millipore-Germany) pH 4.5 was added to 10 mL of pooled serum. The solution was stirrered with dropwise addition of 0.7 mL caprylic acid (Merck Millipore, Germany) (16,17). The solution was centrifuged for 15 min at 5000 ×g and supernatants (containing IgG) was dialyzed with phosphate buffered saline (Merck Millipore, Germany) pH 6.3 for 16 h. For ammonium sulfate precipitation, 10 mL of saturated (NH_4_)_2_SO_4_ (Merck Millipore, Germany) was added to 20 mL of pooled serum drop by drop to reach final 34% saturation. Serum was kept at 4°C while stirring for 2 h and centrifuged for 10 min at 1000 ×g, then the supernatant was discarded (18,19). The precipitation was dissolved in 10 ml phosphate buffer (pH 6.3).

### 3.4.Ion Exchange Chromatography

DEAE-cellulose (10 g; Sigma Aldrich, Germany) dry gel washed with distilled water and left at 4°C for 16 h to remove small particles. The swollen gel was suspended in 0.5 M HCl (Merck Millipore, Germany) for 30 min and was filtered and washed with distilled water.The gel was suspended in 0.5 M NaOH (Merck Millipore, Germany) for 30 min (20) and washed with phosphate buffer (pH 6.3) 5×. The gel was packed into a XK 26/20 column (GE Healthcare, Sweden) with a 300 cm.h^-1^ linear velocity (26.5 mL.min^-1^) using preparative HPLC system (Waters, USA). The packed column was 85 mm bed height and 45 mL bed volume. The column was equilibrated by 3 column volume (CV), 0.07 M buffer phosphate pH 6.3 (19,21) at 225 cm.h^-1^ linear velocity (20 mL.min^-1^). Sample was loaded into the column at 150 cm.h^-1^. Linear flow rate of 13.3 mL.min^-1^ was applied to separate the adsorbed impurities.

### 3.5. Protein Concentration and Purity Assays

Total protein concentration was determined by Bradford assay (22) using bovine serum albumin (Merck Millipore, Germany) as standard. The purity assay was performed by 12.5 percent poly acrylamide gel under reducing condition (23) using 15 mA power supply and 200 kDa unstained protein ladder (Fermentas PageRuler™ SM0661, Germany). The gel was stained with Coomasie blue (Merck Millipore, Germany) and a 30% methanol and 10% glacial acetic acid solutions (both from Merck Millipore, Germany) were used as distaining solution. The distained gel was photographed to analyze purity and the intensity by a gel doc system (Thermo Fisher Inc., Germany).

### 3.6. Western Blot Analysis

The antigens were transferred from SDS-PAGE gel to nitrocellulose membrane (Millipore, Germany) at 45 mA for 45 min. The nonspecific binding sites were blocked by membrane incubation in a 5% BSA (bovine serum albumin) solution (Merck Millipore, Germany) for 30 min. The membrane was washed 3 times with phosphate buffered saline pH 7.2 (Merck Millipore, Germany). Purified antibody in a 1:100 dilution was added to the membrane and incubated for 2 h. The membrane was washed for 2 h with phosphate buffer and incubated with protein A peroxidase (Sigma Aldrich, Germany) as secondary antibody in a 1:1000 dilution. The nitrocellulose membranes were exposed to 15 mL of substrate solution (Thermo Fisher Inc., Germany) for 5 to 15 min. Test was repeated using commercial antibody (Roche, Switzerland).

### 3.7. ELISA (Enzyme-Linked Immunosorbent Assay)

In commercial ELISA kit assay, Erythropoietin specific antibodies have been precoated onto 96-well plates but in this research the purified antibody was coated onto 96 -wells micro plate. The assay was performed according to the manufacturer’s instructions (Roche, Switzerland). Briefly, Standards and test samples including different intermediate samples from different API production steps (Pasteur institute, Iran) were added to the wells along with a biotinylated Erythropoietin detection antibody and the microplate was then incubated at 22°C. Following washing with wash buffer a Streptavidin- horseradish peroxidase conjugate (HRP) was added to each well, incubated at 22°C and unbound conjugates were then washed away using wash buffer. TMB (3, 3’, 5, 5’ - Tetramethylbenzidine) ELISA Peroxidase Substrate was then added and catalyzed by HRP to produce a blue color product that changes to yellow after addition of an acidic stop solution. The density of yellow coloration is directly proportional to the amount of Erythropoietin in plate. The micro plates were read at a wavelength of 450 and 630 nm (24-26). Commercial EPO determination kit (Roche, Switzerland) was used for comparison and the Minitab software (version 17) was used for data analyzing.

## 4. Results

**Figure 1:**
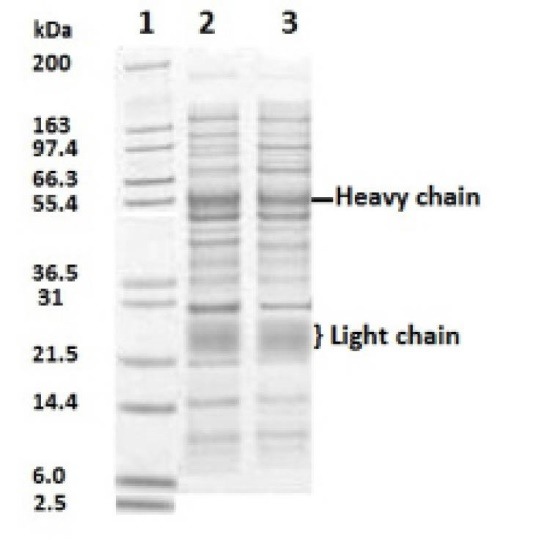
SDS-PAGE results of Immunized rabbits’ serums. Lane 1: marker. Lanes 2 and 3: Serums of two immunized rabbits after injections. Heavy chain was detected in approximately 50 kDa and the light chain migrated as a smear band between 24 and 29 kDa.

**Figure 2:**
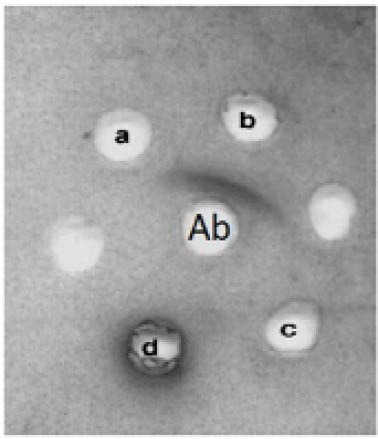
Gel diff usion and agglutination to show specifi c antibody- antigen interaction. Ab: produced antibody against rh-EPO, a: interferon, b: EPO antigen, c: Hbs antigen and d: streptokinase. The archline formation, indicating the antibody- antigen interaction was observed only at EPO antigen site.

**Figure 3:**
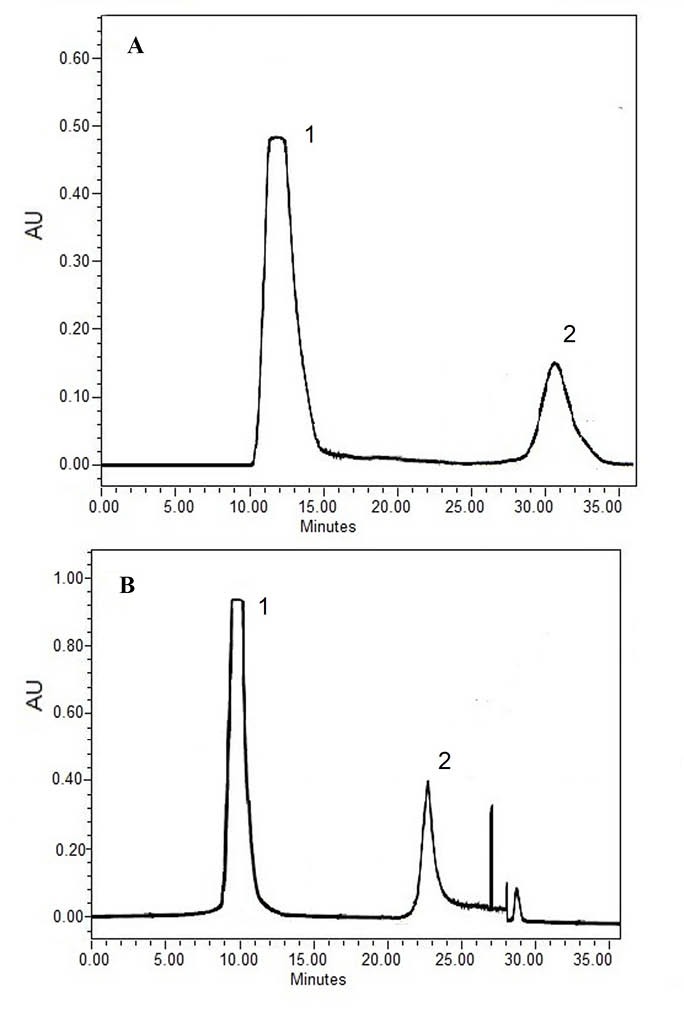
Ion- exchange chromatograms of antibody purification. Ion exchange chromatography of caprylic acid (A) and ammonium sulfate (B) precipitated samples. The fi rst peaks (peak 1) of both 3-a and 3-b fi gures depict fl ow throughs containing target antibody. T he second peaks are related to the column regenerations in which column was washed with 1 molar NaCl solution and all other contaminant attached strongly to the resin were displaced by high concentration of negative ions of Cl–, thus disrobed.

**Figure 4:**
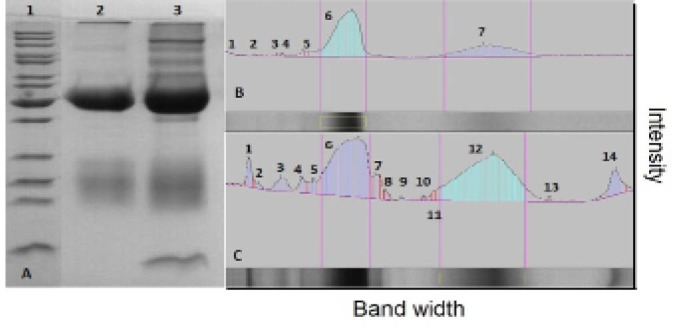
Purity analysis of the fl ow-through peaks of ion exchange chromatography. (A): After caprylic acid (lane 2) and ammonium sulfate (lane 3) precipitation, Lane 1: marker 200, 150, 120, 100, 85, 70, 60, 50, 30, 25, 20 and 15 kDa. (B): Lane 2 analysis, 6^th^ and 7^th^ bands (heavy and light chains) are 63.7 and 33.9 % of total area. (C): Lane 3 analysis, 6^th^ and 12^th^ bands are 36.4 and 43.6%. The purity of antibodies after ion exchange chromatography, obtained from caprylic acid was higher (97%) than ammonium sulfate precipitation method (80%).

Antibody production in the serum of immunized rabbits was checked 2 weeks after injection by SDS-PAGE. Both heavy and light chains were detected in approximately 50 and 25 kDa (Fig. 1). However, the light chain migrated as a smear band between 24 and 29 kDa (27). Agglutination (Table 1) and Ouchterlony tests during the injection and blood sampling were used as initial confirmatory tests to assess the specificity of the antigen and antibody. The gel diffusion method (Fig. 2) for 4 different antigens revealed that the specific antigen- antibody interaction was only detected by EPO antigen. Antibody was purified from serum by either caprylic acid or ammonium sulfate. The purified fractions were dialyzed against phosphate buffer and applied onto DEAE- cellulose ion exchange chromatography. IgG was eluted in the flow through effluent of the column without any absorption to the resin (Figs. 3 and 4). Peaks labeled 1 in 3-a and 3-b refer to the flow through containing the target antibody.

**Table 1:** The agglutination test for different serum antibody dilutions.

**Serum antibody dilutions**	**Agglutination results**			
	**T= 0 (before antigen injection)**	**T=14 day (2 weeks after first antigen injection)**	**T= 28 day (2 weeks after 2nd injection)**	**T= 56 day (2 weeks after last injection)**
direct	-	+	+	+
1:20	-	+	+	+
1:40	-	+	+	+
1:80	-	+	+	+
1:160	-	-	+	+
1:320	-	-	+	+
1:640	-	-	-	+

**Figure 5:**
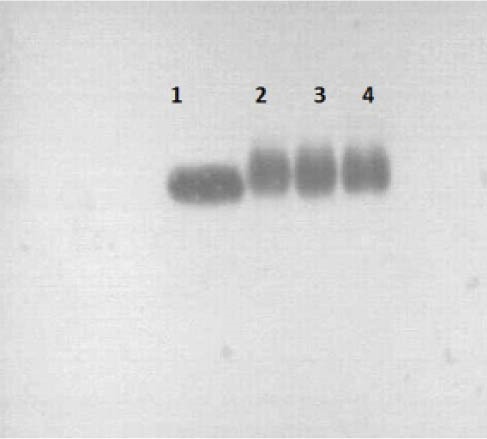
Western blot analysis of EPO antigens with purified antibody in comparison with commercial antibody. Lane 1: detected EPO by Commercial antibody, Lanes 2 - 4: detected EPO by in-house purified antibody.

**Figure 6:**
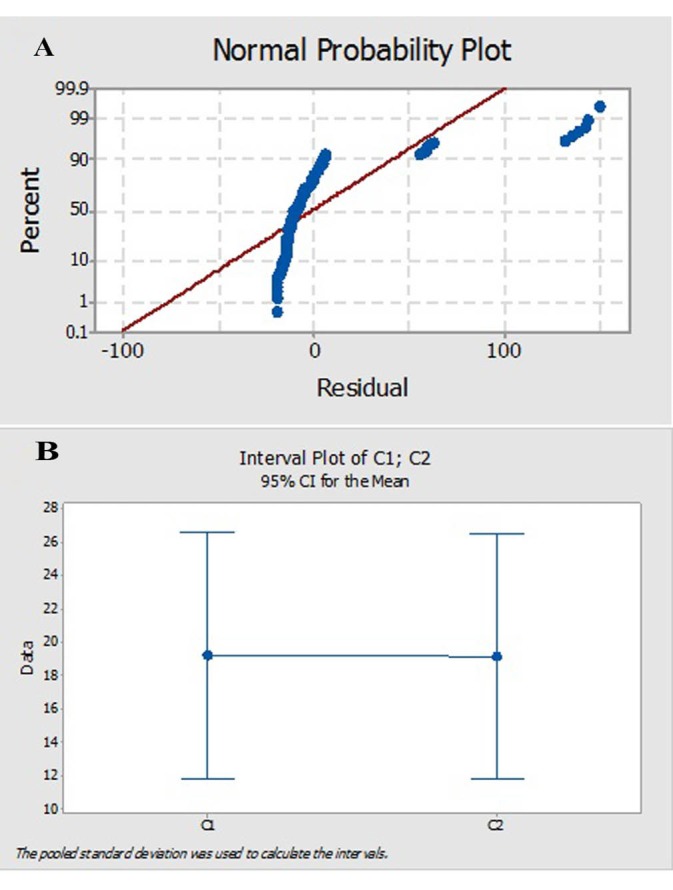
Statistical analysis plots: (A) Normal probability plot and (B) Standard deviation interval plots for purified antibody (C1) and commercial kit (C2) ELISA assay results. Data did not have normal distribution and no diff erence was observed between the means of C1 and C2.

The reaction of purified antibody to the EPO antigen was similar to the standard antibody as demonstrated by western analysis (Fig. 5). Data from ELISA of 24 bio-pharmaceutical samples, different concentrations of erythropoietin, from different API production steps and one negative control were normalized (Table 2) The points (average of each samples) in normal probability plot (Fig. 6A) did not form a straight line, i.e., no normal distribution. The Kolmogorov-Smirnov statistics of both purified antibody and commercial kit were smaller than 0.001. The average data obtained by purified antibody and commercial kit were 19.181 and 19.168, respectively (p < 0.998), indicating non-significant deifference between ELISA assays. The interval plot of the ELISA assays (C1: purified antibody and C2: commercial kit) showed that the means were indifferent (Fig. 6B). The specificity and sensitivity of purified antibody were checked in comparison with commercial kit (Table 3) according to following equations (28,29):

**Table 2:** Comparison of ELISA results of EPO concentrations using commercial kits and produced antibodies.

EPO concentrationsin different samples								
**Samples**	**Antibody test results (µg. mL** ^-1^ **)**				**Commercial kit test results (µg mL** ^-1^ **)**			
**No.**	**1** ^st^ **run**	**2** ^nd^ **run**	**3** ^rd^ ** run**	**Average**	**1** ^st^ **run**	**2** ^nd^ **run**	**3** ^rd^ ** run**	**Average**
1	11	10.3	12	11.1	12.9	12	13.4	12.8
2	21.8	22.3	20.9	21.7	23.4	22	24.5	23.3
3	5.1	4.7	5	4.9	4.4	5	5.5	5
4	6.2	6.9	5.8	6.3	5.3	6.2	6.3	5.9
5	15.5	14.8	14.2	14.8	17.5	18	16.9	17.5
6	4.1	4.8	5.1	4.7	5.4	6.5	5	5.6
7	25	23.9	23.5	24.1	23.1	25.5	24.1	24.2
8	9.1	8.5	8.7	8.8	7.5	7.2	6.1	6.9
9	6	5.1	6.8	6	4.8	4.5	3.9	4.4
10	3.5	3.9	3.1	3.5	5.2	5.7	6	5.6
11	75.2	78.2	77	76.8	82	79.2	80	80.4
12	21.2	19.5	21.1	20.6	19.1	17.8	20	19
13^a^	169.1	161.8	162.9	164.6	155.5	150.8	158.9	155.1
14	13.5	14	13.5	13.7	14.1	13.8	13.1	13.7
15	18.9	18.2	17.8	18.3	18.2	17.3	18.9	18.1
16	11.9	12.5	12.3	12.2	12.3	14	13.2	13.2
17	7.1	6.8	7.8	7.2	5.5	6.8	6.1	6.1
18	5.3	5.8	5	5.4	4.9	4.4	4.3	4.5
19	2.1	1.8	1.6	1.8	1.5	1.8	1.7	1.7
20	16.5	14.8	15.5	15.6	18.9	19.5	17.8	18.7
21	12.1	11	12.9	12	10.5	11	11.6	11
22	9.3	8.3	8.5	8.7	11	10.2	9.7	10.3
23	8.6	9.5	8.1	8.7	8.8	7.9	9	8.6
24	8.6	7.5	7.8	8	7.3	7.3	8.2	7.6
25	0.02	0.05	0.01	0.03	0.03	0.02	0.02	0.02

a: Concentrated API solution to check the high level detections

**Table 3:** Comparison of the sensitivity and specificity of commercial kits and antibodies.

**Parameters for calculating selectivity and specificity**		
**Factors**	**Purified antibody**	**Commercial Kit**
False Positive (FP)	0	0
True positive (TP)	24	24
False negative (FN)	0	0
True negative (NT)	1	1
Sensitivity and specificity	100%	100%

Equation 1: Sensitivity = TP/ (TP+FN)

Equation 2: Specificity = TN/ (TN+FP)

Where

TP: true positive, TN: true negative, FP: false positive, FN: false negative.

## 5. Discussion

To overcome the difficulties of using commercial ELISA and western blot kits for immunological identification of erythropoietin in different intermediate process steps of EPO API production, we tried to produce highly purified in-house polyclonal antibody against erythropoietin. According to optimized immunization guidelines, for rabbit immunization, 50-1000 µg antigen is needed and it is necessary to conjugate small polypeptide antigens (< 10 kDa) to a large protein carrier but for larger protein antigens (30-50 kDa), conjugation is not necessary but will enhance the immunogenic response (30). MI. (2007) in addition to using Freund’s adjuvant, combined EPO antigen covalently to BSA (12) and managed toenhance the antibody production in comparison to using sole EPO antigen/ Freund’s adjuvant (31).

In this work proper immunization was achieved just using 70 µg of EPO antigen + Freund’s adjuvant. Ouchterlony tests showed that antibodies were produced in high level. Two different precipitation methods with ammonium sulfate and caprylic acid followed by anion exchange chromatography with DEAE Cellulose Fast Flow resin were discussed. These precipitation methods were used widely in antibody purification strategies with relative good purity (16,17,32-35). However, Mariam *et al*. (2015) reported 90-94% rabbit IgG purity by using 37-45% ammonium sulfate saturation followed by a mixed mode chromatography (32). The results of this research depicted that purified antibodies obtained from caprylic acid had a higher purity (97%) than 34% saturated ammonium sulfate precipitation method (80%); probably due to the reason that most of the serum proteins except immunoglobulin G with short chain fatty acids can precipitate by caprylic acid at pH 4.2-4.5 (27). In a similar work for purifying antibodies from equine plasma, using sequential precipitation with ammonium sulfate and caprylic acid higher antibody purity was reported by 0% ammonium sulfate and 4% caprylic acid (35). In another research for purifying recombinant antibody from Chinese hamster ovary (CHO) supernatant, caprylic acid/PEG precipitation was competitive with affinity chromatography (16). In ion exchange chromatography of both precipitation method, the antibody was detected in flow through peaks and all albumin content were separated in regeneration peaks. Serum albumin had the iso-electric point of 4.7 (21 and thus at pH 6.3 of equilibration absorbed to the anionic resin . In contrast, rabbit IgG with pI 8.6 (36) has a positive net charge at pH 6.3. Thus, it was eluted in flow through effluent with no absorption to column. Insufficient salt precipitation of the other globulin type blood proteins with the iso-electric points more than 6.3 (for example hemoglobin with iso electric 6.8) could explain insufficient ammonium sulfate precipitation since they could have positive charge and pass the resin without any absorption. Finally, the purified antibody obtained from caprylic acid precipitation- ion exchange chromatography was evaluated to be used as in house antibody in ELISA assay. The purified antibody used as an in-house antibody was able to detect erythropoietin antigen as sensitive and specific as commercial kit. It is to be mentioned the achieved results could be used on other recombinant products.

## Footnotes

Implications for Health policy/practice/ research/ medical education: Biotechnologist in biopharmaceutical production field or biochemical engineers may use the results of presented research.

Authors’ Contribution: All authors have participated equally.

Financial Disclosure: There is no conflict of interest.

Funding/Support:

Pasteur Institute of Iran has provided financial supports.
